# The Multiple Perspectives Approach to Understanding Sexual-Economic Exchange

**DOI:** 10.1007/s10508-025-03259-3

**Published:** 2025-11-01

**Authors:** Norbert Meskó

**Affiliations:** Department of Cognitive and Evolutionary Psychology, Faculty of Humanities and Social Sciences, Institute of Psychology, University of Pécs, Ifjúság Utca 6, Pécs, 7624 Hungary

**Keywords:** Prostitution, Sex work, Human sexuality, Sexual economics

## Abstract

Sexual-economic exchange is a multifaceted phenomenon shaped by biological, psychological, social, and economic factors. This paper examines sexual-economic exchange—including commercial sex and transactional intimacy—through a multidisciplinary lens, integrating perspectives from evolutionary psychology, sexual economics, and the social sciences. Evolutionary models frame these exchanges as adaptive strategies emerging from reproductive asymmetries and resource transfer dynamics. Historically, the institutionalization of private property and male-dominated societies fostered conditions for sex-for-resources arrangements, with prostitution representing an institutionalized and often stigmatized variant. Psychological approaches explore how some individuals arrive at sexual-economic exchange through pathways shaped by early adversity, cognitive patterns, and social marginalization. However, for many, sex work represents a conscious and strategic choice shaped by broader life circumstances and constrained opportunities. Sexual economics theory contextualizes these dynamics within market principles, positing that sexual access functions as a valued resource predominantly regulated by women in heterosexual interactions. Gender asymmetries in sexual desire and resource provision shape mating strategies and intrasexual competition, with societal norms reflecting market dynamics. Efforts to eliminate or liberalize sexual-economic exchange have yielded mixed outcomes, as seen in diverse historical and policy contexts. The persistence of such exchanges underscores the influence of socioeconomic inequality and biopsychological predispositions. This paper advocates for a multiple perspectives approach, integrating the biopsychosocial model, systems theory, and evolutionary psychology to provide a holistic understanding of sexual-economic exchange. This framework is not only conceptually integrative but also practically useful for informing research, improving support services, and guiding evidence-based policy.

## Introduction

Sexual-economic exchange is a controversial phenomenon—often referred to as prostitution or sex work—is commonly understood as providing sexual access to women in return for monetary or other resources. However, due to its complex and multifaceted nature, we adopt a broader definition in the present study. Merriam-Webster (n.d.) defines prostitution as ‘the act or practice of engaging in sexual activity for payment.’ In this broad sense, the term encompasses not only monetary exchanges but also other material or resource-based interactions. As a colloquialism, prostitution likewise refers to a social interaction that is simultaneously sexual and economic in nature (O’Connell Davidson, 1998). This dual nature complicates its classification as a cultural category, as societies often conceptualize and regulate sexual and economic relations through distinct normative frameworks. In this paper, we propose a multidimensional framework for interpreting sexual-economic exchange that is not only conceptually integrative but also practically applicable to research, support services, and policy contexts.

While the present review focuses on the female form of prostitution—defined as women providing sexual services primarily to male clients—this emphasis reflects the predominant global pattern in sex work. The vast majority of sex workers worldwide are women, and clients are overwhelmingly men, regardless of the sex or gender identity of the provider (Baumeister et al., 2001; Platt et al., 2018; Sanders, 2008; UNAIDS, 2014). Although specific dynamics may vary across gender identities, many of the psychological, economic, and evolutionary mechanisms discussed—such as transactional sexuality, stigma, early trauma, and market asymmetries—are also relevant, with contextual modifications, to male and transgender sex workers.

Given the complexities of this phenomenon, the present theoretical review pursues a twofold aim. First, we provide an overview of approaches within the social sciences, psychology, evolutionary theory, and economics that address the roots, functions, and effects of prostitution and sex work. Second, we aim to foster cross-disciplinary dialog by proposing an integrative framework that bridges terminological and paradigmatic gaps, enabling a fusion of theories and a more comprehensive understanding of the phenomenon.

### Prostitution and Sex Work

Over the past decades, a scientific and public debate has emerged between advocates for and opponents of the term prostitution. Opponents of the term emphasize two main arguments. First, its pejorative connotation potentially marginalizes and socially excludes those labeled as prostitutes (Vanwesenbeeck, 2017), depriving them of access to healthcare, social security, and legal protection. Second, the term implies victim blaming, disregarding structural and social factors that drive individuals into the sex market (Sanders, 2008). Victim blaming undermines efforts to address abuse and exploitation. In line with Leigh’s (1997) proposal, others advocate for using the terms sex work and sex worker instead (e.g., Barry, 1995; O’Connell Davidson, 1998; Weitzer, 2012).

Conversely, critics of the term sex work argue that it normalizes and depoliticizes sex trafficking (Farley, 2004; Jeffreys, 2009; Raymond, 2013). By defining sexual service provision as work, it risks obscuring the exploitation and coercion often present (Barry, 1995). Moreover, the term sex work downplays the structural violence and gender inequalities inherent in the sex industry and trivializes the victimization of those involved in prostitution (Farley, 2004). Benoit et al. (2019a, 2019b) highlight that many prostitutes enter the sex market due to economic constraints rather than free choice, a reality inadequately captured by the term sex work.

Some feminist authors (Bindel, 2017; Jeffreys, 2009) argue that sex work terminology overlooks the patriarchal structures and systemic violence shaping the context of prostitution, thus failing to support women’s emancipation. Additionally, critics (Ekman, 2013; Scoular, 2004) warn that the terminology homogenizes diverse forms of sexual services and related experiences, obscuring distinctions between voluntary and coercive participation, which hinders effective interventions.

The proponents and opponents of sex work terminology propose fundamentally different views on the phenomenon. Advocates for the term sex work (e.g., Alexander & Leigh, 1987) emphasize the recognition of sex workers’ rights, better legal protection, and improved working conditions, particularly in regions where public health risks such as HIV are prevalent (Shannon et al., 2018). By contrast, critics (Bindel, 2017; Moran, 2020) argue that normalizing prostitution as sex work overlooks its harmful societal implications and the need for structural change to protect women’s rights and promote equality.

**Fig. 1:**
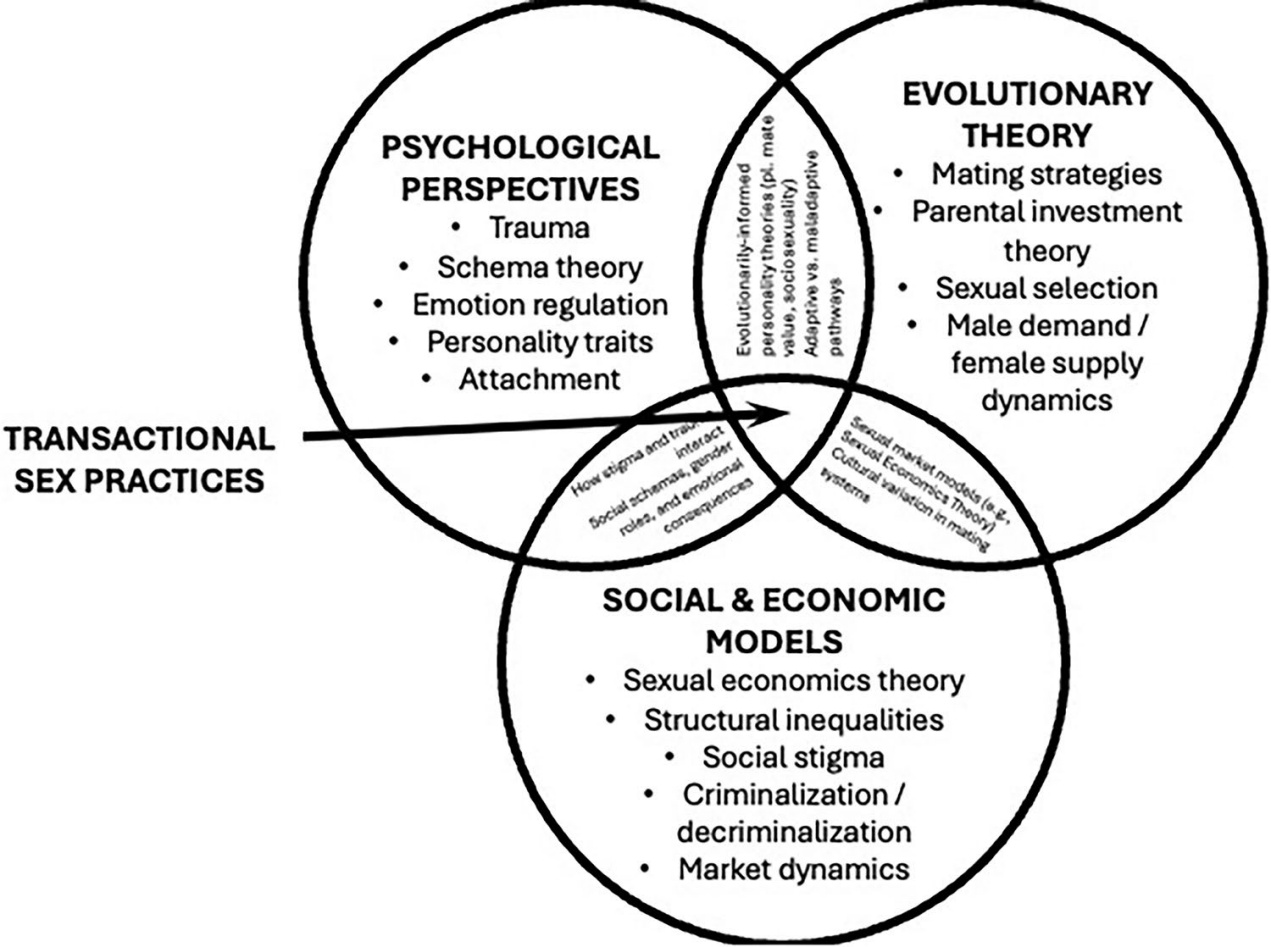
Integrative Framework of Psychological, Evolutionary, Social, and Clinical Approaches to Prostitution and Sex Work. This schematic diagram illustrates the multidimensional nature of prostitution and sex work as understood through four overlapping paradigms: psychological, evolutionary, social, and clinical. Each circle represents a domain of theory and evidence, with key constructs placed within or across domains depending on their primary theoretical anchoring. The central area, labeled “Transactional Sex Practices,” represents the core phenomenon that all four approaches aim to explain or interpret. Overlapping areas highlight concepts or mechanisms that are jointly addressed by two or more paradigms (e.g., trauma, stigma, agency, or adaptive trade-offs), reflecting the need for cross-disciplinary integration in future research.

This ongoing debate highlights the complexity of prostitution as a societal phenomenon. A multidisciplinary approach is essential to fully understand its dynamics and consequences. Existing analyses, such as those by Benoit et al. (2019a, 2019b), provide valuable insights but often overlook psychological perspectives. Building on these, we enumerate the major approaches in the social sciences, present psychological perspectives, elaborate on evolutionary theories, and integrate these with the sexual economics theory to propose the multiple perspectives approach to prostitution (see Fig. 1 for an integrative overview).

Throughout this review, we use both the terms “prostitution” and “sex work.” While “sex work” has gained traction in contemporary discourse for its inclusive and nonjudgmental connotation, we intentionally retain “prostitution” in many parts of the manuscript to reflect historical, legal, and theoretical contexts where that term remains dominant. Our usage is deliberate: The term “sex work” is appropriate when referring to professional frameworks that address the rights, safety, and occupational health of those involved in commercial sex (e.g., in medical, legal, or harm reduction contexts; see Benoit et al., 2018). However, the overuse of the term can also function as a linguistic strategy to normalize or liberalize prostitution as a profession, potentially obscuring the ethical, psychological, or structural vulnerabilities inherent in many transactional sexual arrangements. Research has shown that individuals engaged in transactional sex often do not identify as sex workers, particularly when such exchanges occur in informal, culturally embedded, or economically constrained contexts (Roach et al., 2025). Moreover, the semantic shift from “prostitution” to “sex work” may inadvertently impose a professionalized framework onto behaviors experienced as coerced, exploitative, or emotionally ambivalent (Drouin & McCarthy, 2024). For this reason, we aim to balance terminological inclusivity with conceptual clarity and theoretical precision.

### Discourses in the Social Sciences

In the social sciences, the theorization of, and research on prostitution are based on three major paradigms (Weitzer, 2012). First, the legal-moral paradigm primarily focuses on the social control of prostitution. Second, the gender equality paradigm argues that prostitution is perpetuated by sexual coercion. Third and finally, the free choice paradigm emphasizes, among others, women’s right to physical self-determination. Detailed knowledge of these paradigms is important because their axiomatic assumptions, research questions, terminologies, methodologies and conclusions are not only influential in the social sciences, but they also pervade public discourses on prostitution and sex work (as presented in the previous section). Furthermore, since certain fields of psychology (e.g., social psychology) are affiliated with the social sciences, the below detailed paradigms also influence psychological research.

#### The Legal-Moral Paradigm

The conception of social control of prostitution is based on the moral position that prostitution is a harmful and condemnable activity. Most historical records on prostitution reflect this moral stance (Bernstein, 2007; Bullough & Bullough, 1987; Jeffreys, 2008; Walkowitz, 1980). Historically, one of the earliest approaches to prostitution is the legal-moral paradigm. In this paradigm, prostitution is associated with notions such as criminality and organized crime (Phoenix, 2009). According to this narrative, prostitution poses a threat to society, of which prostitutes themselves are helpless victims (Weitzer, 2007). The legal-moral perspective primarily focuses on regulation, especially regarding criminal law. A related approach considers prostitution as a condemnable activity in religious terms (Outshoorn, 2004). That is, the legal-moral paradigm emphasizes the importance of social control in the conceptualization and treatment of prostitution.

In terms of social control as a form of political power, five major models have been proposed in the social sciences (Immordino & Russo, 2012). First, the *laissez-faire model* is a liberal approach to regulation, which is generally characteristic to large cities with high crime rates, where law enforcement struggles with a lack of capacity and resources (Weitzer, 2012). Considering these circumstances, law enforcement deliberately refrains from intervention (Hubbard et al., 2003). This attitude is characteristic to countries and cities with relatively intense sex tourism, such as Canada, Costa Rica, Cuba, Denmark, Ethiopia, India, Israel, Italy, Malawi, and Portugal, for example (Bandyopadhyay & Khan, 2003; Cabezas, 2009).

Second, the control model focuses on consistent law enforcement, which is generally dependent on a coordinated cooperation between local governments and the courts (Hubbard, 1998). According to Reynolds (1986), the containment of prostitution in a city or district is usually initiated by the local community, who express disapproval of sexual service provision in so-called tolerance zones within their living environment. This situation usually characterizes small towns or middle-class districts of larger towns (Matthews, 1993). In response to community pressure, the local police contain prostitution in areas out of direct contact for the citizens (Scoular & O’Neill, 2007). For local governments, the only legitimate form of control is provided by bans and sanctions.

Third, the regulation model is generally characteristic to cities and regions with substantial rates of legalized prostitution, including escort services, call-girl networks, and brothels (Scoular & O’Neill, 2007). In such cities and regions, access to the market is controlled by licensing. Licensed prostitutes are usually obliged to pay taxes and meet certain health standards. Since there are participants on both the supply and demand side who choose not to observe the regulations under opportune circumstances, the legalized market typically coexists with an illegal market (Weitzer, 2017). As a consequence, the state or federal government takes measures to contain the illegal market. Such a system is operated in Austria, Bolivia, Germany, Greece, Mexico, Senegal, and Turkey, among others (Outshoorn, 2004).

Fourth, the zone model integrates the above three models (Sanders & Laing, 2017). Adult citizens have access to sexual services in designated red-light districts, which enables the authorities to maintain a laissez-faire model within the designated zones, while exercising strict control outside zone boundaries. Illustrative American examples are the French Quarter in New Orleans and the Sunset Strip in Los Angeles (Katz, 2010), while a similar system is operated in the Dutch capital Amsterdam. A characteristic implication of this model is that prostitutes are forced to work in zones lacking adequate protection and control, thus they are exposed to a high risk of victimization by clients and procurers. Moreover, their isolated situation exposes them to discrimination and stigmatization as criminals, which potentially violates their fundamental rights. For example, they may be forced to relinquish child custody as a result of their criminalized status (Scoular & O’Neill, 2007).

In some communities, prostitutes are subject to fines or severe penalties. Fifth and finally, the deterrence model (Durlauf & Nagin, 2011; Nagin, 2013; Webb, 1980) is a popular approach among traditional criminologists, who usually endorse the view that the prospect of severe punishment (deterrence) is an effective means of control over criminal conduct. However, the implementation and operation of this model are highly resource-demanding, since it requires well-organized operational police forces, capable prosecutors and courts, and a coordinated detention and probation system (Brewer et al., 2006).

In sum, the legal-moral paradigm proposes that prostitution can be suppressed by adequate social control, provided that the employed model fits the social conditions (e.g., structure and size of the local administrative area, demographic characteristics of the population) under which it is implemented (Weitzer, 2010). It should be noted, however, that the legal-moral paradigm is frequently criticized for disregarding the social problems associated with the systemic discrimination against prostitution and prostitutes, on which the model is essentially based.

#### The Gender Equality Paradigm

The gender equality paradigm is based on the tenet that female emancipation depends on women’s ability to liberate themselves from male-dominated power structures (e.g., Coy, 2012). From this perspective, gender equality remains unattainable as long as women’s sexual interactions with men reflect systemic imbalances in power and autonomy. Prostitution is interpreted as the most extreme case of gender-based oppression, where sexuality is not viewed as a reciprocal interaction between equal partners but as a commodified transaction serving male sexual gratification. In this view, individuals engaged in prostitution are understood primarily as victims of structural and interpersonal oppression, even if their actions may conflict with social norms (Bell, 1994; Farley et al., 2017). Adverse childhood experiences—including physical, sexual, and emotional abuse—are frequently cited as contributing to later involvement in prostitution (Dodsworth, 2012). Accordingly, prostitution is framed not as a product of genetic predisposition or moral decline, but as a manifestation of sexually oppressive patriarchal systems (Miller & Schwartz, 1995).

This position is promoted by anti-prostitution feminists, who argue that prostitution as an institutionalized occupation carries severe long-term consequences for women’s physical and mental health (Kramer, 2004; Spector, 2006). Pathogenic factors include exposure to client and third-party violence, drug-related vulnerabilities, and elevated risk of HIV infection. Furthermore, women in prostitution are often subjected to social exclusion and stigmatization, being perceived as deviant from prevailing cultural norms (Aronson et al., 2001).

Another fundamental tenet of the gender equality paradigm is that women are often coerced into prostitution through manipulation, threat, abuse, or lack of viable alternatives (e.g., Raphael et al., 2010). From this perspective, what is sold on the sex market is not labor in the conventional sense, but the capacity to endure sexual violation; prostitution is thus conceptualized as a form of sexual violence (Farley, 2018). Proponents of this paradigm advocate for collective efforts aimed at dismantling coercive circumstances and supporting those vulnerable to entering prostitution (Bell, 1994).

#### The Free Choice Paradigm

Another feminist approach to prostitution is the free choice paradigm, which emphasizes women’s oppression in society and the restriction of their individual rights inherent in oppressive practices (Davidson, 1995). Advocates for this paradigm are involved in feminism as far as they strive to gain recognition for women’s rights (to independence, financial autonomy, sexual self-determination, abortion, same-sex relationships, and sexual service provision; Zatz, 1997). Free choice is understood as the basis for the protection of women’s individual rights, whose restriction is equivalent to the infringement of women’s social status, while justifying such restrictions as serving to protect women from themselves amounts to denying equal rights to them (Jolin, 1994). This paradigm has motivated rhetorical innovations, as a result of which the term prostitution, associated with deviancy in public discourses, has given way to the term sex work, for example (Alexander, 1998).

According to the proponents of the free choice paradigm, prostitution is understood as an institutionalized form of hierarchical sexual relations, in which male dominance is normalized and women’s structural vulnerabilities are reproduced. In this framework, individuals involved in prostitution are considered to be exploited due to their position at the intersection of systemic inequalities characteristic of neoliberal capitalist societies—including class, gender, and race (e.g., Benoit et al., 2019a, 2019b). From this perspective, the principle of free choice affirms that sex workers should have the same right to engage in their form of labor as do physical laborers or professional athletes. The paradigm draws from the postmodern feminist view that reality is rooted in individual or group experiences, and that truth is inherently contextual, fluid, and socially constructed (Dietz, 2003).

Public debates on prostitution typically involve proponents of the above presented three paradigms, including opinion leaders, representatives of interest groups, and the general public. Furthermore, these three paradigms are thematized in various disciplinary conceptualizations of prostitution proposed by lawyers, politicians, psychologists, historians and sociologists. Beyond theoretical formulations, the free choice paradigm is also reflected in the activism and documentation of sex worker-led organizations. For example, the Global Network of Sex Work Projects (NSWP), staffed and led by sex workers, articulates community-based understandings of sex work as labor and emphasizes rights-based advocacy (NSWP, 2021a, 2021b, 2024).

### Psychological Approaches

In psychology, two closely interrelated research streams have given rise to two broad theoretical approaches to understanding prostitution. The victimological approach emphasizes the psychopathologies associated with the physical, emotional, and sexual abuse that frequently characterize the lives of those involved in prostitution (e.g., Mancuso & Postlethwaite, 2020). By focusing on the impacts of victimization, this approach seeks to illuminate the underlying vulnerabilities and maladaptive coping mechanisms resulting from such experiences. In contrast, the cognitive approach is rooted in the concept of schemas—cognitive structures that regulate perception and thought processes (Beck, 1976). This perspective examines how maladaptive schemas, often formed in response to adverse experiences during childhood, influence behavior and psychological well-being in adulthood. The following sections provide a detailed exploration of these two complementary frameworks, highlighting their contributions to a deeper understanding of the psychological underpinnings of prostitution.

#### The Victimological Approach

Victimology, a specialized field within criminology, focuses on understanding the experiences of individuals who have been harmed by crime or violence, including their characteristics, coping mechanisms, and the broader social dynamics of victimization (Fattah, 1991; Karmen, 2012; Walklate, 2007). It explores how personal histories and environmental risk factors shape vulnerability to future victimization and evaluates social systems intended to support recovery (Doerner & Lab, 2011).

In a developmental victimological framework, Pereda (2015) identifies various forms of childhood abuse—physical, emotional, and sexual—that contribute to long-term psychological vulnerabilities and maladaptive coping strategies. These factors, including attachment difficulties, substance use, and low self-esteem, have been associated with increased susceptibility to engaging in prostitution. Updegrove and Muftic (2019) further emphasize that cumulative childhood adversities are linked to a greater risk of revictimization and adult exposure to violent crime, particularly among women involved in sex-for-resources exchanges (Benoit et al., 2015).

Matthews (2015) offers a structural perspective on victimization, highlighting how social, economic, and legal forces shape the life circumstances of women involved in sex-for-resources exchanges. Similarly, Gnaim-Mwassi et al. (2024) examine retrospective accounts of Arabic women, finding that histories of sexual abuse, cultural pressure, and lack of social support often interact to constrain their life options.

Consistent evidence indicates that women involved in prostitution report elevated levels of depression, anxiety, and trauma-related symptoms compared to other women (MacLean et al., 2018; Millan-Alanis et al., 2021; Wondie et al., 2019). They also face higher rates of substance dependence and HIV risk (Surratt et al., 2005; Ulibarri et al., 2010; Su et al., 2014). These mental health outcomes have often been linked to early sexual abuse and chronic developmental stressors (Abramovich, 2005; Bagley & Young, 2009), which may disrupt interpersonal functioning in adulthood.

Decades of empirical research have shown a strong association between childhood sexual abuse and increased vulnerability to engaging in sex-for-resources exchanges later in life (Vaddiparti et al., 2006; Wilson & Widom, 2010; Wu et al., 2010). These findings do not imply that all women involved in prostitution have been victimized or lack agency; rather, they highlight one frequently observed pathway. In this context, victimological frameworks contribute to understanding how structural inequality and developmental trauma intersect, without denying the diversity of women’s motivations, circumstances, or self-perceptions within sex work.

This framework also aligns with research on pornography performers, who are sometimes viewed through the same lens of trauma and damage. For instance, Griffith et al. (2013) examined the so-called “damaged goods hypothesis” in the context of adult film actors, finding that perceptions of emotional and psychological dysfunction are often attributed to this population. While empirical findings are mixed, extending victimological thinking to pornography performers points to a broader cultural narrative linking sex work with psychological vulnerability.

Another concept relevant to victimological perspectives is the notion of the “whorearchy”—a term used to describe the informal hierarchy among different types of sex workers, including street-based workers, escorts, cam models, pornography performers, and sugar babies (Hubbard & Sanders, 2008; Levy, 2015). This hierarchy not only reflects differences in working conditions and degrees of agency but also mirrors the varying levels of social stigma assigned to each category. For example, street-based sex workers are often perceived as the most marginalized and stigmatized, while those working through online platforms or in high-end escorting may experience greater autonomy and social acceptance—though still subject to moral judgment and exclusion (Cunningham & Kendall, 2011). Recognizing this internal stratification adds valuable nuance to discussions of stigma, psychological vulnerability, and structural violence within sex work.

#### The Cognitive Psychological Approach

The cognitive psychological approach to understanding prostitution centers on the concept of schemas—cognitive structures that regulate perception and thought processes (Beck, 1976). These schemas operate at three distinct levels of mental representation. First, negative automatic thoughts are beliefs that are easily triggered in certain situations, often leading to a distorted perception of reality. Second, dysfunctional attitudes encompass maladaptive responses to various scenarios and are less accessible to conscious awareness. Third, self-schemas—deeply ingrained representations of the self—are rooted in early childhood and shape how individuals perceive their relationships with themselves and their social environment (Romanowska & Dobroczyński, 2020).

Schemas play a mediating role in linking childhood trauma to psychopathological symptoms in adulthood (Gibb et al., 2001; Rekart et al., 2007). Often referred to as core beliefs or early maladaptive schemas, these cognitive structures develop in response to unmet emotional needs during childhood, such as secure attachment, self-regulation, emotional expression, and realistic boundaries (Bach et al., 2018). Typically operating outside of conscious awareness, these schemas influence information processing, leading to distorted perceptions of reality (e.g., Kopcsó & Láng, 2014). Negative parental attitudes and adverse social environments during childhood act as external risk factors, increasing vulnerability to psychopathologies.

Research has consistently linked early maladaptive schemas to various psychological outcomes, including attachment issues (Mikulincer et al., 2003; Kaya & Aydin, 2021), childhood trauma (Basso et al., 1999; Mącik, 2021), mental disorders such as female sexual dysfunctions (Mancuso & Postlethwaite, 2020), bipolar disorder (Panagiotopoulos et al., 2023), interpersonal difficulties (Thimm, 2013), substance use (Shorey et al., 2012, 2013), risky sexual behavior or hypersexuality (Gilliland et al., 2011; Roemmele & Messman-Moore, 2011).

Sexual self-esteem, defined as emotional reactions to one’s own sexual thoughts, feelings, and behaviors, is another key construct in this framework (Alimoradi et al., 2022). It is closely tied to the ability to form satisfying sexual relationships (Weber et al., 2024). Higher sexual self-esteem is associated with greater control over one’s sexual desires and experiences (Oattes & Offman, 2007), while lower sexual self-esteem is linked to sexual dysfunction and psychological distress (Tayebi et al., 2021). Research suggests that all forms of childhood abuse, including sexual abuse, can significantly reduce self-esteem in adulthood, with long-term consequences for sexual functioning and interpersonal relationships (McCarthy & Maughan, 2010; Sachs-Ericsson et al., 2010).

Early maladaptive schemas often stem from childhood experiences of disconnection and rejection (Karatzias et al., 2016). These schemas, such as emotional deprivation, abandonment, mistrust, and defectiveness, reflect unmet emotional needs and contribute to long-term interpersonal difficulties. Victims of childhood sexual abuse frequently exhibit these schemas, which manifest in negative self-perceptions and maladaptive coping mechanisms, such as engaging in high-risk sexual behavior to alleviate anxiety or seek self-esteem boosts (Barnes et al., 2016; Roemmele & Messman-Moore, 2011).

Among women engaged in sex work, maladaptive schemas significantly influence their sexual self-esteem and interpersonal relationships. For example, schemas of emotional deprivation and abandonment are linked to distrust in relationships and a belief in their inability to form meaningful connections (Shareh, 2016). This mistrust often leads to a reduction in social contact, while sexual activity becomes a compensatory strategy for unmet emotional needs (Benoit et al., 2018; Putnam, 2003). Women with such schemas frequently perceive themselves as sexually unattractive or unworthy, further reinforcing their maladaptive beliefs and perpetuating cycles of rejection and isolation (Nasir et al., 2010).

These schemas also explain why women involved in sex work often struggle to maintain healthy relationships and experience difficulties in forming intimacy. Negative experiences during childhood, particularly sexual abuse, impair their ability to trust others and establish secure attachments. Studies highlight how these early experiences are associated with feelings of shame, low self-esteem, and an increased preference for short-term, non-committed sexual relationships (Estévez et al., 2019; Harding et al., 2012; Karantzas et al., 2023).

Prostitutes with low sexual self-esteem are more likely to engage in unstable sexual relationships, reflecting their maladaptive schemas of emotional deprivation, abandonment, and mistrust (Shareh, 2016). These schemas shape their perceptions of themselves and their relationships, reinforcing cycles of rejection and perpetuating feelings of inadequacy. Such findings underscore the profound and lasting impact of childhood experiences on adult sexual behavior and self-perception.

While psychological explanations of prostitution often emphasize the role of early trauma, parental neglect, and attachment disruptions (Wilson & Widom, 2010; van den Berg et al., 2015; Ross et al., 2004), it is important to note that most empirical studies in this domain rely on retrospective self-report data and cross-sectional designs, which limit the ability to draw firm causal inferences (Farley et al., 2004; Moran & Farley, 2019). In addition, the role of heritable traits such as emotional instability, impulsivity, or vulnerability to psychopathology has been underexplored in this context, despite growing evidence on the genetic transmission of risk for personality disorders and emotional dysregulation (Krueger et al., 1996; Livesley et al., 1993; Viding & McCrory, 2019). Recent findings from a study involving young adult women (Meskó et al., 2025a) indicate that individuals with greater openness to engaging in sugar relationships—without actual involvement in sex work—exhibit significantly higher levels of maladaptive emotion regulation strategies (as measured by the CERQ; Garnefski et al., 2001), maladaptive personality traits (Krueger et al., 2012), and early maladaptive schemas (Young et al., 2003), particularly in the domains of abandonment, emotional deprivation, social isolation, and impulsivity. These findings support the notion that certain psychological vulnerabilities may precede, and potentially predispose individuals to, the adoption of transactional sexual scripts rather than being mere consequences of engaging in sex work.

Beyond trauma-related vulnerabilities, some studies suggest that specific personality traits—such as high openness to experience, impulsivity, and sensation seeking—may also predispose individuals to consider sex work as an attractive lifestyle option. For instance, Edlund et al. (2023) found that sex workers scored higher in adventurousness, a subfacet of openness, indicating greater comfort with novelty and non-conformity. Similarly, anecdotal accounts from sugaring studies report that some women enter transactional sexual relationships because they are already engaging in frequent casual sex and view compensation as a pragmatic extension of existing behaviors (Griffith et al., 2013). Broader literature also supports the association between impulsivity, sensation seeking, and risky or non-normative sexual behavior (Roberts et al., 2014; Seto & Lalumière, 2010). However, these motivational pathways do not necessarily contradict victimological or developmental accounts. On the contrary, traits such as impulsivity or sensation seeking may not only facilitate entry into high-risk sexual environments but also amplify susceptibility to coercion, boundary violations, or gradual erosion of agency—particularly in the absence of stable attachment models or emotional regulation skills. Thus, while internal motivations may initially play a role in the decision to engage in sex work, they can coexist with—and even potentiate—the psychological mechanisms that lead to cycles of exploitation or self-stigmatization. Recognizing this duality offers a more nuanced understanding of how personal dispositions and structural vulnerabilities interact in the context of sex work.

### Evolutionary Explanations

The ethological framework proposed by Tinbergen (1963) provides a dual-level approach to understanding animal behavior by distinguishing between proximate and ultimate causes. This distinction offers a deeper and more comprehensive understanding of human behavior as well. Psychological research typically emphasizes proximate causes, examining the immediate physiological, hormonal, and neural mechanisms underlying behavior, as well as the environmental stimuli or events that trigger these responses. Developmental psychologists also focus on proximate causes when modeling the relationships between new behavioral patterns and the hypothetical psychological processes driving them throughout an individual’s development. In contrast, explanations based on ultimate causes delve into the evolutionary origins and adaptive functions of psychological mechanisms, aiming to understand why certain behaviors evolved and how they contribute to reproductive success (Bereczkei, 2000).

While many traditional psychological approaches to prostitution focus on proximate causes, such as the immediate triggers or underlying mechanisms of the behavior, evolutionary perspectives shift the focus to ultimate causes (e.g., Burley & Symanski, 1981; McGuire & Gruter, 2003; Salmon, 2008). These explanations seek to uncover the evolutionary origins and adaptive significance of prostitution as a phenomenon. Existing evolutionary theories on prostitution are diverse, as they address different aspects of the behavior. Rather than being competing frameworks, these theories should be viewed as complementary pieces of a larger puzzle, each contributing unique insights to create a more holistic understanding of the phenomenon (Meskó, 2014).

From an evolutionary psychological perspective, it is important to distinguish between ultimate (adaptive) functions and proximate (psychological and emotional) mechanisms of sexual exchange (Tooby & Cosmides, 1992). In romantic pair-bonding contexts, sexual and emotional exchanges are typically not consciously calculated; rather, they are mediated by evolved proximate mechanisms such as romantic attraction, attachment, and idealization (Eastwick et al., 2014; Zeifman & Hazan, 2008). These mechanisms facilitate long-term commitment and cooperation by fostering strong emotional bonds between partners.

In contrast, sex work typically involves explicit transactions with limited emotional involvement and minimal activation of these evolved attachment mechanisms. This divergence in underlying motivational architecture helps explain the psychological and behavioral differences between commercial sex and romantic sexual interactions, even when the surface structure of exchange appears similar.

#### Exchange of Sex for Resources in Human Mating

In non-human animals, sexual accessibility and resource provision often form the basis of transactional interactions between the sexes (Kamimura et al., 2021; Lewis & South, 2012). These interactions exhibit distinct sex-based patterns: individuals of one sex (typically females), who invest more heavily in offspring, provide mating opportunities in exchange for resources offered by the other sex (typically males) (Krebs & Davies, 1993; Petersen, 2022). Sexual reproduction is inherently energy-intensive, requiring the transformation of environmental resources into the energy necessary for reproduction (Del Giudice et al., 2015; Noë & Hammerstein, 1994; Schowalter, 2022). These sexual exchanges serve as adaptive mechanisms for energy sharing, benefiting the reproductive success of both sexes. For many species, tailoring these behaviors to their ecological niche is essential for survival (Fisher & Pruitt, 2020).

Examples of such exchanges are widespread across species. Male scorpionflies (*Bittacus apicalis*) provide prey to their mates, while females allocate their energy to egg production. Interestingly, females often select mates based on the size of the prey offered (Thornhill, 1976). Similarly, female Adélie penguins (*Pygoscelis adeliae*) engage in mating with males from other colonies in exchange for stones used to construct nests, which significantly influence reproductive success (Hunter & Davis, 1998). Among primates, male pygmy chimpanzees (*Pan paniscus*) are known to share food with females they have previously mated with (de Waal, 1995). Male macaques (*Macaca fascicularis*), particularly those with lower social status, spend considerable time grooming females to gain sexual access, a behavior less frequently required of dominant males (Gumert, 2007).

Like non-human females, women must invest substantial energy in reproduction due to the biological demands of pregnancy, breastfeeding, and child-rearing (Savard et al., 2021). Social support during these stages critically enhances infant survival rates (Elsenbruch et al., 2007). Sexual transactions, in various forms, are also prevalent in human societies (Kwena et al., 2017; Ringdal, 2006). In exchange for sexual accessibility, men may provide women with love, commitment, respect, attention, physical protection, material goods (e.g., luxury items, property), or opportunities such as career advancement (Baumeister & Vohs, 2004). In long-term mating contexts, such transactions often involve men taking on sustained responsibility for resource provision in return for exclusive sexual access to their partner (Baumeister & Vohs, 2012).

In certain pre-industrial societies, such as the polygynous Kipsigis in Kenya, men are required to pay a bride price to marry (Borgerhoff Mulder, 1990). Wealthier men, able to afford higher bride prices, often marry younger and more fertile women, thus achieving greater reproductive success (Borgerhoff Mulder, 2000). Moreover, wealthier men can provide better nutrition and healthcare for their families, reducing infant mortality and shortening childbearing intervals (White & Burton, 1988).

Among the Pimbwe people of western Tanzania, cultural norms permit relatively unrestricted marriage, divorce, and remarriage for both genders (Borgerhoff Mulder & Ross, 2019). However, remarriage tends to benefit women more than men, as women frequently seek new partners to improve their financial circumstances. This behavior likely reflects an adaptive strategy in response to the variable quality of land owned by Pimbwe men, given the critical role of material resources in infant survival (Borgerhoff Mulder, 2009).

Short-term sexual transactions, characterized by the exchange of sexual accessibility for material compensation without emotional attachment, are also common (Burley & Symanski, 1981; Salmon, 2008; Symons, 1979). According to sexual strategies theory (Buss & Schmitt, 1993, 2019), prostitution represents an extreme form of short-term mating strategy. Men, in this context, prioritize cues signaling women’s immediate sexual availability, while women respond to signals of men’s willingness to share resources directly.

#### The Behavioral Ecological Approach

Meskó et al. (2014) argue that institutionalized prostitution is not solely driven by the interests of clients and sex workers. To gain a deeper understanding of this phenomenon, they extended empirical research to examine the attitudes of women in long-term relationships toward their partner’s infidelity. The findings suggest that, in certain evolutionary contexts, women may have gained adaptive advantages by tolerating their partner’s infidelity when it was confined to transactional sex (e.g., with a stranger) rather than an emotionally invested affair (e.g., with a lover).

Behavioral ecology emphasizes the measurable reproductive outcomes of behavior rather than its psychological underpinnings (Cronk, 1991). It explores how adaptive behaviors are shaped by ecological and social environments and examines whether these behaviors correlate with reproductive success (Barta et al., 2002; Cockburn, 1991). A core principle of behavioral ecology is that natural selection pressures organisms to optimize resource acquisition and utilization to maximize genetic fitness. This optimization can take two primary forms: (1) investing in individual physical growth and fitness or (2) prioritizing reproductive success through mate choice, parental care, and kin relations (Bereczkei, 2000; Sng et al., 2018).

However, resource availability is finite, forcing individuals to allocate time and energy strategically. Investments in one area, such as physical fitness, inherently limit resources available for reproduction. Because reproduction is energetically costly, natural selection has favored decision-making mechanisms that optimize resource allocation. These mechanisms are rooted in the brain’s ability to process environmental cues and provide alternative behavioral responses based on a cost–benefit analysis of survival and reproductive investments. Natural selection rewards behaviors that yield the greatest net reproductive benefit in a given environment (Borgerhoff Mulder, 1992).

Human ancestors were under selective pressure to accurately evaluate environmental factors critical to survival and reproduction. Although contemporary environments differ significantly from those of the evolutionary past, the brain centers responsible for adaptive decision-making remain attuned to similar stimuli. As a result, individuals continue to adopt strategies for resource distribution that enhance genetic fitness (Bereczkei, 2000).

The adaptive support theory (Meskó & Láng, 2013; Meskó et al., 2012, 2014) builds on these principles, proposing that women’s reproductive success in the evolutionary past was more constrained by a lack of resource investment from their partner than by sexual infidelity. Consequently, women evolved to be more attuned to emotional infidelity, which poses a greater threat to resource investment, than to purely sexual infidelity (Campbell & Ellis, 2005). However, another evolutionary force—the threat of infection—also shaped responses to infidelity. Sexual contact carries risks of transmitting parasites and sexually transmitted diseases (Mackey & Immerman, 2000). These two evolutionary pressures—avoiding abandonment due to emotional infidelity and avoiding infections through sexual infidelity—pulled women’s mate-retention strategies in opposite directions (Hock & Fefferman, 2012; Neuberg et al., 2011). The adaptive support theory suggests that women’s responses to their partner’s infidelity reflect a trade-off mechanism that balances these competing risks while accounting for current environmental threats (Gangestad & Simpson, 2000).

From the perspective of reproductive fitness, a male partner’s infidelity with a woman involved in prostitution presents lower abandonment risks but higher infection risks compared to an emotional affair with a former partner. Meskó et al. (2014) examined how varying environmental threats, such as infection risk versus abandonment risk, influence women’s attitudes towards different types of infidelity. Their research in Hungary, a society with a developed healthcare system, found that women were more likely to tolerate their partner’s infidelity with a woman involved in prostitution than with a former romantic partner. Participants associated a higher likelihood of emotional commitment with love affairs involving ex-partners and the lowest likelihood of emotional commitment with sexual encounters involving a sex worker. Conversely, the risk of infection was perceived as highest in scenarios involving sex workers and lowest in emotional affairs with former partners.

When asked which scenario they would find most tolerable if temporarily unable to engage in sexual contact with their partner, women showed mixed responses. They were least lenient toward the scenario involving a prostitute (due to infection risk) and most lenient toward infidelity with a stranger (to mitigate abandonment risk). These findings suggest that women employ flexible, context-dependent strategies to minimize potential losses associated with their partner’s infidelity, rather than rigidly adhering to a single approach.

This approach underscores the adaptive complexity of human decision-making in response to infidelity (Buss & Schmitt, 1993, 2019). Women’s behavioral strategies reflect an evolutionary balance between competing pressures, tailored to specific environmental threats, and aimed at optimizing reproductive success. Such findings highlight the intricate interplay of ecological, social, and psychological factors in shaping human mating behavior.

#### Runaway Selection

Forrai (2010) posits that runaway selection (Fisher, 1930; Ridley, 1994) may be a significant evolutionary mechanism contributing to the emergence of prostitution.^1^ The original concept of runaway selection, first introduced by Fisher (1930), addresses a Darwinian paradox: how do selection processes preserve traits that appear disadvantageous for survival but are highly attractive to potential mates (e.g., the extravagant tail plumage of male peacocks)? Darwin (1871/1963) resolved this tension by suggesting that both the ornamentation and the preference for it are genetically transmitted. He hypothesized that later-evolving species developed an "esthetic sense," which favored the co-selection of such traits.

Fisher (1930) extended this idea, proposing a genetic link between ornamental traits and the preference for them. Initially, these traits were associated with higher reproductive fitness, ensuring their positive selection. Over time, however, the continued preference for increasingly exaggerated traits can exert pressure contrary to natural selection, as these traits may no longer indicate reproductive advantage. This feedback loop between the ornamentation and preference can drive exponential growth in the traits, a phenomenon Fisher described as runaway selection (e.g., Gayon, 2010). Although this theory offers compelling explanations for certain phenotypes, its empirical validation remains challenging due to the difficulty of observing both the triggering processes and their underlying genetic mechanisms (Andersson & Iwasa, 1996).

Forrai (2010) extends runaway selection to human evolution, suggesting that ecological and social changes have mediated the development of specific human preferences and behaviors through both cultural and genetic pathways. The foundational psychological traits of modern humans—thoughts, emotions, desires, and behaviors—are products of evolutionary processes spanning hundreds of thousands of years (Barkow et al., 1995). During the Pleistocene, early humans lived as hunter-gatherers in tribal societies, and their physiology and behaviors adapted to this lifestyle. However, two transformative revolutions—the Neolithic Revolution (10,000–14,000 years ago) and the Industrial Revolution (3–4 centuries ago)—profoundly altered the socioecological environment (Borrell et al., 2015; Glenn, 2004).

These societal changes introduced new social challenges, including the institutionalization of private property and the emergence of class structures. Agricultural societies cultivated and amassed economic surplus, necessitating male physical labor and coordinated patrilineal systems to manage and protect these resources (Flannery, 1972; Smith, 1995). This shift reinforced patriarchal structures characterized by dominance and aggression (Smuts, 1995), relegating women to roles centered on reproduction and domesticity. By contrast, hunter-gatherer societies emphasized interdependence and relative gender equality (Larsen, 2003). Anthropological studies of tribal societies, such as the Aché of Paraguay, highlight the essential contributions of women’s labor, with women supplying 20–40% of daily caloric intake through foraging (Kaplan et al., 2000).

Cultural shifts also shaped men’s parental investment strategies. In agricultural societies, where wealth and resources were inheritable, men prioritized resource acquisition over direct child-rearing, as economic contributions outweighed parental involvement in reproductive success (Katz & Konner, 1981). Conversely, in resource-scarce hunter-gatherer societies, men often devoted more time to child-rearing, as direct investment in offspring offered greater reproductive advantages in these contexts (Fortunato, 2012; Rotkirch, 2018). These contrasts illustrate how socioecological conditions influence gender roles and mating strategies.

The objectification of women and the valuation of their reproductive capacity also find roots in agricultural societies (Forrai, 2010). Polygyny remains prevalent in many patriarchal agrarian societies, where women are valued as assets based on their reproductive potential. Among the Kipsigis of Kenya, for example, bride price—a cultural mechanism of male intrasexual competition—requires men to pay livestock or money for marriage (Borgerhoff Mulder, 1990). Wealthier men often acquire multiple wives, which intensifies competition and leaves many men unmarried (Hartung et al., 1982). Furthermore, wealth enables these men to secure younger, more fertile brides, thus enhancing their reproductive success (Borgerhoff Mulder, 2000). This dynamic also improves familial health outcomes, as wealthier men provide better nutrition and healthcare, reducing infant mortality and shortening childbearing intervals (White & Burton, 1988). These observations underscore the enduring interplay between economic conditions and reproductive strategies across human societies.

Forrai (2010) integrates the theory of runaway selection into evolutionary explanations of prostitution, illustrating how biological and cultural factors jointly shape human reproductive behaviors. By linking preferences and behaviors to socioecological transformations, runaway selection offers a valuable perspective on the persistence of prostitution as a bio-psychological phenomenon deeply embedded in human evolution.

One potentially illuminating contrast between evolved female mating preferences and the dynamics of sexual-economic exchange concerns the issue of choice. From an evolutionary perspective, female mate choice plays a critical role in sexual selection, shaping not only individual reproductive outcomes but also long-term, species-level trends (Buss, 1989; Gangestad & Simpson, 2000). In prostitution, however, the opportunity to exercise mate choice is often constrained by economic pressures, safety concerns, and the commercial nature of the interaction. Nevertheless, research suggests that many female sex workers retain some degree of agency in selecting or refusing clients, often based on perceived hygiene, manners, personal attraction, or risk of violence (Sanders, 2005). These decision criteria partially overlap with dimensions known to guide female mate preferences more broadly, such as health, kindness, and resource availability (Li et al., 2002).

Importantly, sex work is itself stratified along social, economic, and contextual lines—ranging from street-based or brothel-based workers with limited autonomy to high-end escorts or financially supported companions, such as so-called “sugar babies,” who may not self-identify as sex workers at all (Bernstein, 2007; Monto & Milrod, 2014). As in other areas of social life, the degree of personal agency—including the ability to select clients or partners—is often greater for individuals in higher-status positions. This stratification mirrors broader social hierarchies and further complicates the question of how sexual selection pressures manifest in contemporary transactional contexts.

### The Sexual Economics Approach

As outlined in the preceding sections, psychological theories of sexuality intersect significantly with other disciplines, particularly political science and biology. Political science, especially within feminist studies, often frames sexual behavior through the lens of male dominance and female victimization. In contrast, biology, particularly evolutionary psychology, examines the reproductive contingencies underlying human sexuality. Extending this interdisciplinary scope, Baumeister and Vohs (2004) introduced an economic perspective on sexuality.

At first glance, applying economic principles to human sexuality may seem counterintuitive, as money—the foundation of economic studies—and sexuality are characterized by distinct dynamics (Baumeister et al., 2017). Money derives its value from its purchasing power, independent of its holder or specific use. Sexuality, on the other hand, carries value that is deeply subjective and shaped by the emotional and social context of actual or potential partners. Moreover, factors like a partner’s sexual history or offspring from prior relationships influence its perceived worth (Symons, 1979). Despite these differences, sexual economics reveals striking parallels between financial markets and human mating behaviors, offering a conceptual framework for understanding how individuals negotiate sexual relationships and mate choices.

Sexual economics theory (Baumeister & Vohs, 2012) applies the logic of market economies to heterosexual relationships, conceptualizing sexual interactions as transactions. In this framework, women are viewed as “sellers” and men as “buyers,” based on reproductive biology: Sexual access to women holds greater value due to their higher reproductive investment (Trivers, 1972). Women’s higher energy costs in reproduction, including pregnancy and childcare, make their sexual availability a highly sought-after resource across cultures.

This gender-based asymmetry is reflected in men’s higher sexual drive and their tendency to seek sexual relationships earlier and with fewer prerequisites than women (e.g., Kenrick et al., 1990; Simpson & Gangestad, 1991). For example, men report higher frequencies of sexual thoughts, fantasies, and desires across various measures of sexual behavior (Baumeister et al., 2001). According to sexual economics, this imbalance grants women greater control over sexual exchanges, allowing them to negotiate terms that maximize their benefits (Waller & Hill, 1951).

Crucially, sexual economics does not narrowly define these exchanges as monetary transactions, as in prostitution. Instead, it highlights a broader spectrum of exchanges, where women may acquire non-material resources such as love, commitment, social status, or professional opportunities (Béné & Merten, 2008). Historical examples reveal that, prior to industrialization, men often assumed long-term responsibility for providing resources in exchange for exclusive sexual access to their partners. The theory avoids moral judgment, focusing instead on how sexual exchanges operate as socioeconomically driven transactions (Vohs & Baumeister, 2015).

In recent years, newer forms of transactional intimacy—such as sugar dating or “sugaring”—have gained attention. In these arrangements, younger individuals (typically women) engage in romantic or sexual relationships with older, wealthier partners (typically men) in exchange for material benefits, financial support, or lifestyle access (Birkás et al., 2020; Ipolyi et al., 2021; Láng et al., 2021). While many sugar dating participants distance themselves from the label of prostitution (Mixon, 2019), the underlying structure of sexual-economic exchange often closely mirrors more traditional forms of sex work. Sugaring exemplifies how evolved psychological sensitivities—particularly to cues of resource availability and provisioning—can be expressed within new socioeconomic frameworks (Meskó et al., 2025a, 2025b). Its popularity highlights the adaptive flexibility of mating-related behaviors, while also raising ethical and legal debates over exploitation, agency, and blurred boundaries between sex work and consensual intimacy (Meskó et al., 2024; Motyl, 2012).

The market-driven nature of sexual relationships also shapes intrasexual competition. Women compete primarily by enhancing their sexual attractiveness relative to other women (Wang & Griskevicius, 2014), while men focus on signaling their resource-providing capabilities (Hennighausen & Schwab, 2014; Sundie et al., 2011). These competitive dynamics underscore the differing motives underlying male and female choices in sexual relationships: Men prioritize physical attractiveness and sexual pleasure, whereas women seek partners who can provide material or social resources (Baumeister & Vohs, 2004).

Sexual market dynamics are subject to the same principles of supply and demand that govern traditional markets. When women outnumber men in a given population, liberal sexual norms emerge, lowering the “price” of sexual access (Schmitt, 2005). Conversely, when men outnumber women, sexual norms become more conservative, reflecting increased competition and higher standards for male contributions (Griskevicius et al., 2011). This alignment of sexual norms with demographic imbalances exemplifies how economic principles shape sexual interactions.

The Sexual Economics Theory also refines evolutionary perspectives by emphasizing the context-dependence of sexual behaviors (Buss, 2003). Men adapt their strategies based on market conditions: For instance, in environments where women are scarce, men adopt more restricted sociosexual behaviors to align with the prevailing dynamics (Schacht & Mulder, 2015). These findings demonstrate how both genders adjust their mating strategies in response to fluctuating supply and demand within the sexual economy.

Ultimately, Sexual Economics Theory provides a nuanced framework for understanding human mating behavior, integrating biological imperatives with sociocultural and economic influences. The theory highlights how individuals exchange a variety of resources—not limited to material wealth—for desirable psychological traits and sexual partners. By encompassing biological, social, and economic factors, sexual economics broadens our understanding of the intricate dynamics underpinning human sexual interactions (Whyte et al., 2019). Recent formal economic models have further extended this framework by conceptualizing sexual exchanges as utility-maximizing strategies embedded within human capital development. These models suggest that sexual behavior may form part of broader life planning, shaped by educational attainment, career aspirations, and long-term socioeconomic goals (Carroni et al., 2025).

## Discussion

### The Problem of Terminology

Given that the previously addressed debates on the terms prostitution vs. sex work are not confined to terminology per se, but they express the differential ideological commitments of scientific paradigms and discourses, it is unlikely that the proponents of one or the other perspective will give up their preferred terminology any time soon. It is worth considering, therefore, that the term exchange of sex for resources offers an ideologically impartial alternative to both prostitution and sex work, which are historically eroded and politically value-laden notions. In this sense, exchange of sex for resources is a descriptive term (see Meskó et al., 2024), and thus it may better meet the purposes of scientific discourse in the international literature. A recent review has argued that the term sexual-economic exchange may offer a conceptually neutral alternative to ideologically contested terms such as sex work or prostitution (Crankshaw & Freedman, 2023; see also Tabet, 2012).

Importantly, sex worker-led organizations such as the Global Network of Sex Work Projects (NSWP) have developed comprehensive resources that articulate community-driven perspectives on terminology and stigma. Their Terminology Statement and Guide (NSWP, 2024) advocates for respectful, non-stigmatizing language rooted in lived experience, while their Briefing Paper on the Consequences of Misinformation (NSWP, 2021a) highlights the harm caused by inaccurate or pathologizing narratives. These resources underscore the importance of engaging directly with sex worker voices when discussing conceptual and linguistic frameworks. These perspectives are further developed in other NSWP materials addressing systemic barriers to justice (NSWP, 2020), participation in public life (NSWP, 2021b), and the impacts of anti-rights movements on sex workers globally (NSWP, 2022).

While this article adopts sexual-economic exchange as its primary descriptive term, it also recognizes the terminological diversity present in the literature and in social discourse. Accordingly, earlier terms such as “prostituted woman” may appear in cited texts, but this manuscript refers to them only when reflecting the original conceptual framework of the source. These variations are not intended to promote or oppose any particular ideological stance but rather to maintain fidelity to the original context of empirical findings and theoretical arguments.

This pragmatic approach reflects the understanding that language is not merely a neutral vehicle for description, but also a site of ongoing contestation shaped by political, moral, and epistemological commitments. By acknowledging this complexity while maintaining a primary commitment to conceptual clarity, the term sexual-economic exchange aims to provide a functional and ideologically neutral alternative suited for scientific analysis across disciplinary boundaries. The use of different terms throughout this manuscript is thus deliberate, transparent, and grounded in the logic of contextual appropriateness rather than ideological alignment.

While this paper focuses on structural, psychological, and evolutionary dimensions of sexual-economic exchanges, it acknowledges that individual experiences vary widely, and that for many women, sex work may represent a conscious and strategic choice shaped by complex life circumstances.

### The Problem of Eliminating Prostitution

The exchange of sexual access for resources is deeply intertwined with human mating strategies (Buss, 2003; Symons, 1979; Trivers, 1972). From an evolutionary perspective, women’s openness to sex-for-resources exchanges (also referred to here as sexual-economic exchange) can be seen as an adaptive trait that historically enhanced reproductive success in both long- and short-term mating contexts. In early agricultural societies, dominated by male power structures, the rise of private property and its institutionalization after the Neolithic Revolution (Bocquet-Appel & Bar-Yosef, 2008; Diamond, 1997) created an environment where sex-for-resources transactions became a cultural norm for long-term partnerships. Prostitution, however, emerged as an extreme manifestation of short-term relationships. Given the persistence of the bio-psychological foundations—such as men’s preference for sexual variety and women’s capacity for promiscuity—alongside enduring socio-environmental conditions, including male-dominated social hierarchies, prostitution’s prevalence is unlikely to decline substantially in the foreseeable future.

Research has demonstrated that openness to sex-for-resources exchanges is measurable as an attitude, regardless of whether individuals are directly involved in prostitution or sex work (Birkás et al., 2020; Ipolyi et al., 2021; Láng et al., 2021; Meskó et al., 2024). This openness is linked to characteristics such as self-centered sexual motivation, unrestricted sociosexuality, a ludus (game-playing) love style, and socially maladaptive traits like Machiavellianism, subclinical psychopathy, and borderline personality (Birkás et al., 2020; Láng et al., 2021). Additionally, extrinsic motivations further correlate with a tendency toward transactional sexual relationships (Ipolyi et al., 2021). The disproportionately high prevalence of borderline and antisocial personality disorders among women involved in prostitution (Brody et al., 2005; Edwards & Verona, 2016; Tull et al., 2011) suggests that these vulnerabilities may predate involvement in sex work. This hypothesis is supported by findings indicating that childhood abuse significantly increases susceptibility to entering prostitution in adulthood.

While evolutionary and psychological models have repeatedly documented average sex differences in mating preferences—such as men’s relatively greater desire for sexual variety and women’s stronger orientation toward emotionally secure relationships—these patterns are not universal. A subset of women may pursue alternative reproductive or economic strategies that diverge from the majority pattern, including those involving short-term mating or commodified intimacy. Sex work, in this light, may reflect one such contextually shaped alternative, without implying pathology or moral failure.

Women’s inclination to assess potential partners’ willingness to invest materially in relationships appears to be an intrinsic aspect of their mating psychology. Consequently, prostitution is likely to thrive in social environments conducive to short-term sexual relationships. Eliminating prostitution would therefore require addressing the systemic socioeconomic inequalities that sustain it—a task that current knowledge deems unfeasible.

Historical attempts to eradicate either socioeconomic inequalities or prostitution itself have largely failed or produced mixed results. In the Soviet Union, for example, prostitution was officially denied under communist doctrine, which framed it as a remnant of capitalist and class-based societies supposedly eradicated by socialism (Attwood, 1990). Despite this denial, prostitution persisted in various forms within the Soviet Union and the broader Eastern Bloc (Coleman & Sandfort, 2014; Gal & Kligman, 2000). A different approach was adopted in Sweden with the passage of the Nordic Model in 1999, which criminalizes clients purchasing sexual services while exempting sex workers from legal liability (Ekberg, 2004). While this model reportedly reduced visible street prostitution (Kingston & Thomas, 2019; Vuolajärvi, 2019), it also drove the trade into less visible arenas, increasing sex workers’ vulnerability to violence (Levy, 2013; Levy & Jakobsson, 2014; Nanni, 2021). Similar mixed outcomes have been observed in other countries adopting the Nordic Model, including England, Wales, and Northern Ireland (Jang, 2024; McMenzie et al., 2019; Scoular & Carline, 2014). As Levy and Jakobsson (2014) noted, an unintended consequence of this approach was the geographical displacement of demand to neighboring countries with more permissive regulations.

The persistence of prostitution reflects its deep entanglement with fundamental human behaviors and entrenched societal structures, making its complete eradication an unrealistic goal. Efforts to eliminate it often fail to acknowledge the adaptive and systemic forces that sustain it across cultures and eras. Rather than pursuing unattainable absolutes, a more pragmatic approach lies in mitigating harm, addressing inequalities, and fostering environments that prioritize the well-being and autonomy of those involved.

### The Problem of Liberalizing Prostitution

Sexual liberation movements often advocate for the decriminalization and normalization of prostitution, framing these goals within the broader context of individual rights and freedoms (Vanwesenbeeck, 2017). However, such aspirations frequently overlook the profound psychological impacts that sexual liberation may impose on both sex workers and society as a whole.

Walter et al. (2020) argue that women generally favor stable, long-term relationships that secure emotional and material investments, while men are more inclined toward strategies that increase the number of sexual partners. Prostitution, characterized by women frequently changing male partners, conflicts with evolutionary strategies optimized for child-rearing and survival. Although it provides short-term benefits for both clients and sex workers, it often fails to deliver the emotional and material stability offered by long-term relationships and can result in long-term negative consequences for sex workers.

Sex workers commonly experience low self-esteem, exacerbated by the stigma associated with their work (Farley, 2018). Despite the efforts of sexual liberation movements to reduce stigmatization, these self-esteem issues often persist. Recent studies by Gu et al. (2014), Martín‐Romo et al. (2023), and Millan-Alanis et al., (2021) report a high prevalence of depression and suicidal ideation among sex workers, often attributed to the emotional burden of physical and psychological trauma. Leary and Baumeister (2000) posit that self-esteem functions as an internal barometer of social status and esteem. Stigmatization and social exclusion deprive sex workers of status and social support, thereby significantly impairing their psychological well-being.

Mogilski et al. (2020) suggest that societal stigmatization and moral disapproval of promiscuity reflect deep-seated evolutionary dynamics. Individuals are predisposed to endorse behaviors aligned with their life strategies and reject those that conflict with them. Those pursuing a slow life strategy prioritize long-term relationships, invest heavily in parenting, and foster strong emotional bonds. In contrast, fast life strategies involve short-term relationships, higher numbers of sexual partners, and less investment in parenting. Promiscuity, often associated with prostitution, poses a potential threat to slow life strategists by undermining the stability required for enduring relationships and effective parenting.

Recent research (Beattie et al., 2020) indicates that sex workers exhibit significantly higher levels of post-traumatic stress disorder (PTSD) symptoms compared to individuals in other occupations. The frequent exposure to sexual violence and recurrent physical abuse makes sex workers particularly vulnerable to trauma (Vanwesenbeeck, 2017). Tooby and Cosmides (1990) propose that PTSD may function as a survival mechanism, prompting individuals to avoid situations reminiscent of past trauma. The prevalence of PTSD among sex workers highlights the severe and lasting consequences of the maltreatment they endure.

Interpersonal relationships among sex workers often deteriorate due to the stigmatization and social isolation inherent in their work (Gorry et al., 2010). The lack of trust and meaningful social connections frequently undermines their ability to develop and maintain deep relationships (Sanders, 2004). Dunbar (1998) underscores the importance of social networks for survival and reproduction, yet the conditions surrounding sex work erode these critical connections. This perspective sheds light on the psychological toll of social isolation and emphasizes the need for robust support systems to safeguard sex workers’ mental well-being.

Sex workers are also disproportionately exposed to violence and exploitation, as demonstrated by a recent study (Deering et al., 2020). Beyond physical harm, such violence leads to long-lasting trauma that affects mental health and interpersonal relationships. Although efforts to liberalize prostitution aim to empower sex workers and reduce stigma, they often fall short of addressing the systemic inequalities and structural barriers that sustain exploitation and marginalization. Studies underscore the necessity for nuanced, context-sensitive interventions that balance individual agency with societal transformation (Månsson & Hedin, 1999; Platt et al., 2018; Wilson & Nochajski, 2018).

Addressing the complexities of prostitution requires a critical, holistic approach that considers both individual vulnerabilities and broader societal dimensions. Such an approach must aim to provide adequate protection and meaningful support for sex workers while challenging the structural forces that perpetuate their exploitation.

### Theoretical Integration: The Multiple Perspectives Approach

The multiple perspectives approach to prostitution represents a multidisciplinary framework that synthesizes various unidisciplinary theories and explanations for sex work. It is underpinned by three theoretical pillars: the bio-psycho-social model of human sexuality, systems theory, and the evolutionary psychology meta-theory.

The biopsychosocial model of human sexuality offers a holistic lens for understanding sexual health and behavior by integrating biological (e.g., hormones, neurobiology), psychological (e.g., emotions, mental health), and social factors (e.g., social norms, relationships) into a cohesive interpretive framework (e.g., Bancroft, 2009; Heiman, 2002; Hyde & DeLamater, 2016; Levine, 2003; Sims & Meana, 2010). This model underscores the interplay of these dimensions, providing a comprehensive approach to analyzing the factors that shape sexual behavior and its various manifestations.

Systems theory (Von Bertalanffy, 1968) serves as an interdisciplinary framework for examining the structure, dynamics, and interactions within complex systems. A foundational principle of systems theory is that a system is not merely the sum of its parts; instead, it constitutes an integrated whole where emergent properties and behaviors arise from the interactions of its components. The theory distinguishes between physical, biological, social, and abstract systems, making it broadly applicable across domains such as biology, psychology, sociology, and management. Within the context of prostitution, systems theory elucidates how biological and psychological factors—such as genetic predispositions, hormonal regulation, mental health, and psychological states—interact and influence individual behaviors and decisions about sex work (Vanwesenbeeck, 2017).

Social and cultural contexts further shape the dynamics of prostitution, as norms, legal frameworks, economic conditions, and cultural attitudes converge to define its forms and persistence in a given society (Weitzer, 2009). Systems theory provides a comprehensive perspective on how these social and cultural elements interweave with economic and political factors, including labor market conditions, social support systems, and legal regulations, to influence the phenomenon of prostitution (Platt et al., 2018). By framing these interdependent elements as a dynamic and adaptive system, systems theory enhances our understanding of how factors like economic pressure, stigmatization, and legal interventions shape both the lived experiences of those involved and the broader societal responses (Dodsworth, 2014; Krüsi et al., 2016; Levy & Jakobsson, 2014).

The meta-theory of evolutionary psychology offers a robust framework for understanding prostitution through the lens of reproductive and social dynamics (Barash & Lipton, 2001; Buss, 2003; Symons, 1979; Thornhill & Palmer, 2000; Wilson & Daly, 1992). This perspective posits that human behaviors, including violence and exploitation, can function as mechanisms for consolidating power and dominance within social hierarchies (Sidanius & Pratto, 2020). The victimization and exploitation often experienced by sex workers may reflect deeper, historically entrenched dynamics within human societies (Deering et al., 2020). An evolutionary lens contributes to identifying these patterns and developing targeted strategies to mitigate them (McKibbin et al., 2008).

Historically, human behavioral patterns have evolved to maximize reproductive success. Men often increased their genetic fitness through multiple partnerships, thereby enhancing opportunities to pass on their genes, while women prioritized securing stable partners who could provide resources and stability for offspring (McGuire & Gruter, 2003). In modern societies, prostitution persists under economic and social conditions that align with these evolutionary pressures. The interplay between economic constraints and the dynamics of the sexual market influences the prevalence and forms of prostitution. From an evolutionary standpoint, women’s participation in prostitution may be viewed as a strategic adaptation to environmental and economic pressures (Dylewski & Prokop, 2021).

In summary, evolutionary psychology situates prostitution as a complex phenomenon rooted in the interplay between human sexual strategies and modern economic and social environments. Prostitution is not merely a result of individual choices but also a manifestation of behavioral patterns deeply embedded in the evolutionary fabric of human societies.

While the previous sections outlined the individual contributions of each theoretical perspective, it is equally important to consider how these approaches interact to provide a more integrated and systemic understanding of prostitution and sex work. The following section explores the dynamic interplay among these perspectives and illustrates how their integration enhances both theoretical coherence and practical insight.

### Theoretical Integration: Interactions Between Perspectives

Integrating evolutionary psychology, the biopsychosocial model, and systems theory offers a more comprehensive understanding of prostitution and sex work than any single perspective could achieve on its own. Each framework contributes distinct yet complementary insights: Evolutionary psychology illuminates the adaptive mechanisms underlying human sexual behavior; the biopsychosocial model emphasizes the interplay of biological, psychological, and social influences; and systems theory highlights the dynamic interdependencies between individuals and their broader environments.

At the biological level, evolutionary psychology explains how sexual preferences, motivations, and vulnerabilities may have been adaptive in ancestral environments. These include men’s evolved preference for sexual variety and women’s attunement to partner investment—patterns that may inform both normative and transactional sexual behaviors (Buss, 2003; Symons, 1979). However, these tendencies do not operate in isolation. They are shaped by psychological factors such as attachment styles, emotion regulation capacities, and trauma histories (Farley, 2018; Leary & Baumeister, 2000), as emphasized by the biopsychosocial model. These psychological dimensions mediate how individuals respond to environmental stressors and opportunities, including economic hardship and social stigma (Gu et al., 2014; Millan-Alanis et al., 2021).

Systems theory bridges these levels of analysis by demonstrating how evolved predispositions and psychological traits are embedded in and shaped by broader societal structures, such as legal policies, economic systems, cultural narratives, and gender norms (Levy & Jakobsson, 2014; Platt et al., 2018; Vanwesenbeeck, 2017). For example, even if some sexual behaviors originate from evolved reproductive strategies, their contemporary expression in prostitution is influenced by structural inequalities, labor market dynamics, migration patterns, and technological developments such as online escorting. These elements function as interconnected components of a complex system.

Accordingly, the multiple perspectives approach should not be understood as a simple aggregation of theoretical viewpoints, but rather as a layered framework in which causal mechanisms at different levels interact. Evolutionary insights help explain the origins and persistence of sex-for-resources exchanges, psychological models clarify individual experiences and coping strategies, and systems theory situates these phenomena within broader social and institutional contexts.

Figure 1 illustrates this integrative framework by organizing biological, psychological, and sociocultural levels into a dynamic model. It shows how proximate and ultimate factors co-regulate sexual behavior within evolving sociocultural systems. While each perspective retains its distinct conceptual logic, their intersection reveals points of vulnerability, opportunities for targeted interventions, and directions for empirical research.

This integrative approach is not an exercise in theoretical eclecticism but a deliberate effort to achieve explanatory synergy. The multifaceted nature of prostitution calls for such multidimensional theorizing—not only to advance scholarly understanding, but also to inform interventions that are attuned to the real-world interplay between biology, psychology, and systemic forces.

While the theoretical integration of perspectives enhances our conceptual understanding of prostitution and sex work, it is equally important to consider what this integration means in practice. How can researchers, professionals, and policymakers apply these insights in real-world contexts? The following section outlines the practical implications of the multiple perspectives approach, offering guidance on how a systems-oriented, multidisciplinary mindset can inform research, intervention, and decision-making.

### Practical Implications: Applying the Multiple Perspectives Approach in Research, Support Services, and Policy-Making

The multiple perspectives approach is not merely a theoretical construct—it also serves as a practical framework for decision-making across various domains. Its integrative nature is particularly valuable in addressing complex phenomena such as prostitution and sex work, where interventions often fall short due to narrow or fragmented understandings.

For researchers, this approach encourages the development of multi-layered research questions that account for both proximate and ultimate causes, as well as systemic interactions. Rather than focusing exclusively on psychological traits (e.g., impulsivity, attachment style) or structural factors (e.g., legal frameworks, economic disadvantage), scholars are invited to investigate how these dimensions intersect. For example, a study might explore how childhood trauma (psychological), combined with housing instability (socioeconomic), predicts involvement in sex work differently depending on sociosexual orientation (evolutionary predisposition). Under this model, mixed-methods designs and interdisciplinary collaboration are not just valuable—they are essential (Platt et al., 2018; Vanwesenbeeck, 2017).

For helping professionals—including therapists, social workers, and healthcare providers—the multiple perspectives approach emphasizes the need to view sex workers not solely through psychological or victimization lenses, but as individuals embedded in dynamic systems and shaped by evolutionary motives. A systems-informed intervention might simultaneously address emotional regulation difficulties and the social isolation stemming from stigma and legal precarity. At the same time, an evolutionary-informed perspective can help normalize certain behaviors as adaptive survival strategies, reducing moral judgment and enhancing therapeutic alliance (Farley, 2018; Leary & Baumeister, 2000).

For policymakers, the approach underscores the importance of designing legal, economic, and social interventions that reflect a realistic understanding of human behavior. Policies that focus exclusively on criminalization or de-stigmatization, without addressing underlying structural inequalities or psychological vulnerabilities, are likely to yield limited or unintended consequences (Jang, 2024; Levy & Jakobsson, 2014). A theoretically integrated policy framework might combine targeted financial support for at-risk populations (systemic level), trauma-informed mental healthcare (psychological level), and public education campaigns that reduce stigma while acknowledging the complex motivations behind sex work (evolutionary and social levels).

Across all domains, the value of the multiple perspectives approach lies in its ability to conceptualize prostitution across interconnected levels of analysis. It discourages reductionist thinking—whether in the form of biological essentialism, individual blame, or structural determinism—and instead promotes a dynamic, context-sensitive, and ethically responsible understanding of sex work as a complex human phenomenon.

### Conclusions

Prostitution is a deeply entrenched phenomenon that resists simplistic explanations and one-dimensional interventions. The complexity of its underlying mechanisms—spanning biological predispositions, psychological vulnerabilities, and sociocultural dynamics—necessitates a departure from narrow, discipline-specific approaches. Instead, a holistic framework is essential to fully address its roots, manifestations, and broader societal implications.

The persistent interplay between individual-level factors—such as early trauma and maladaptive schemas—and systemic-level forces—including gender inequalities, legal structures, and economic disparities—underscores the necessity of a multidisciplinary perspective. Prostitution cannot be effectively understood or mitigated without integrating insights from evolutionary biology, psychology, economics, and the social sciences. This integrative perspective reveals how structural conditions intersect with psychological and behavioral factors to sustain the phenomenon across cultures and historical periods.

Policy interventions often fail because they isolate specific aspects of prostitution, such as legal status or public health, without addressing the broader systems that perpetuate it. A systemic approach advocates for interventions that simultaneously target individual vulnerabilities and societal inequalities. This includes reducing the stigma faced by sex workers, improving access to mental health care, and mitigating the economic precarity that drives many into transactional sexual relationships.

While this article approaches prostitution from an evolutionary standpoint, it is important to stress that this perspective is descriptive, not prescriptive. The identification of evolved psychological sensitivities to the exchange of sex and resources should not be mistaken for moral endorsement or the naturalization of commercial sex. On the contrary, evolutionary reasoning helps to explain not only how such sensitivities may have been adaptive in ancestral environments, but also how they can be co-opted and exploited in modern contexts. Precisely because humans possess an evolved responsiveness to the strategic use of sex for resource exchange, this mechanism can be manipulated under conditions of inequality, coercion, or trauma. Evolutionary explanations thus offer insight into both the functional origins and the potential vulnerabilities of human sexual behavior—enhancing, rather than diminishing, the ethical and social relevance of the framework.

The multiple perspectives approach exemplifies the value of theoretical and practical integration. By synthesizing the biopsychosocial model, systems theory, and evolutionary psychology, this framework not only enriches our conceptual understanding of prostitution but also provides a roadmap for researchers, clinicians, and policymakers alike. It invites us to move beyond ideological polarization and toward nuanced, evidence-based strategies that acknowledge the complexity of human sexuality while promoting the dignity, agency, and well-being of those affected.

## Footnotes

1 Forrai (2010) speculates that prostitution may represent an extreme by-product of runaway sexual selection, wherein certain male sexual preferences—especially for signs of youth, availability, and exaggerated femininity—become decoupled from adaptive value and instead reinforce cultural institutions that artificially fulfill these preferences.
